# Modulational instability of coupled ring waveguides with linear gain and nonlinear loss

**DOI:** 10.1038/s41598-017-04408-y

**Published:** 2017-06-22

**Authors:** Nguyen Viet Hung, Krzysztof Zegadlo, Aliaksandr Ramaniuk, Vladimir V. Konotop, Marek Trippenbach

**Affiliations:** 1 Advanced Institute for Science and Technology, Hanoi University of Science and Technology, Hanoi, Vietnam; 2 Faculty of Physics, University of Warsaw, ul. Pasteura 5, PL-02-093 Warszawa, Poland; 3 Centro de Física Teórica e Computacional Faculdade de Ciências and Departamento de Física, Faculdade de Ciências, Universidade de Lisboa, Campo Grande, Edifício C8, Lisboa, 1749-016 Portugal

## Abstract

We consider a nanostructure of two coupled ring waveguides with constant linear gain and nonlinear absorption - the system that can be implemented in various settings including polariton condensates, optical waveguides or atomic Bose-Einstein condensates. It is found that, depending on the parameters, this simple configuration allows for observing several complex nonlinear phenomena, which include spontaneous symmetry breaking, modulational instability leading to generation of stable circular flows with various vorticities, stable inhomogeneous states with interesting structure of currents flowing between rings, as well as dynamical regimes having signatures of chaotic behavior.

## Introduction

Ring-shaped structures are a natural framework for studying diverse phenomena in both optics and physics of degenerate quantum gases. For the long time it was used for such applications as ring interferometers, ring lasers^1^, and optical gyroscopes^2^, just to mention a few. More recently, nonlinear microring resonators were proven to be of importance for switches^3, 4^ and lasers^5, 6^, as well as for controlling the dispersion and nonlinearity of waveguides^7^. In the case of atomic Bose-Einstein condensates (BECs) the ring-shaped geometry allows for obtaining persisting superfluid currents and study their interaction with various types of the defects. That is why dynamics of atomic BECs loaded in toroidal traps have been intensively explored experimentally^8–12^ and studied theoretically both in the full three-dimensional toroidal geometry^13, 14^ and within the framework of the reduced quasi-one-dimensional Gross-Pitaevskii equation (GPE) with periodic boundary conditions^15–17^. Rotational dynamics of BECs in dissipative models, was considered in refs 18 and 19 (see also a recent overview paper^20^). Waveguides with ring geometry are also of special interest in the physics of polariton exciton condensates^21^.

In addition to the cyclic geometry, many of the above applications (systems) share another physical property, that is the (cubic) nonlinearity. This makes the mathematical modeling of all of them alike. In optical systems, the Kerr nonlinearity is introduced by the intensity-dependent refractive index of the medium and in the mean-field theory of condensates it appears due to two-body interactions.

What introduces the essential difference in the dynamical behavior of the above mentioned systems is accounting or not for the dissipative effects. In particular, the GPE describing atomic BECs is conservative and therefore they retain a number of integrals of motion (like energy, linear and angular momenta, topological charges of vortices and vortex solitons, etc.) and support families of nonlinear modes characterized by the dependence of the chemical potential on the total number of atoms. Unlike atomic BECs exciton-polariton condensates are open systems^22–24^. They require the gain mechanism compensating losses. In latter case the nonlinear currents, when excited, represent isolated solutions (which in the context of the dynamical systems are called attractors). It is this last type of the excitations that is in the focus of the present paper. They exist at a specific chemical potential (for more extended discussion of the differences between solitons and dissipative solitons see e.g. refs 25 and 26). Here we are interested in the phenomena arising from the coupling of two ring waveguides in the presence of homogeneous linear gain and nonlinear losses. We would like to stress that in optics both settings, conservative and dissipative, are also very common and results of investigations presented below may be applied to optical systems as well.

The paper is organized as follows. In Sec. 1 we formulate the model and introduce its main physical characteristics. In Sec. 2 we consider in detail the types of the homogeneous solutions in the double-ring configuration (Sec. 2.2), their stability and dynamics. Inhomogeneous states are considered in Sec. 3. The outcomes are summarized in Conclusions.

### 1 The model

To formulate the problem, let us assume that in the case of atomic BECs atoms are loaded in a narrow toroidal trap and at the same time are removed from the condensate due to inelastic two-body interactions. Alternatively, one can consider an exciton-polariton condensate whose the field is incoherently pumped^27^ into the systems but also undergoes nonlinear absorption. Yet another setting can be an optical ring resonator where the waveguide is active (say, doped by active impurities and pumped by an external laser) and we include the two-photon absorption. We also assume that the linear gain in all above models is characterized by a positive constant *γ*, and nonlinear losses are characterized by a positive constant Γ, both of which are homogeneous along the waveguide. Then the model describing the field guided in such a ring is described by the dimensionless nonlinear Schrödinger (NLS) equation with linear gain and nonlinear losses

1 $$i{\partial }_{t}{\rm{\Psi }}=-{\partial }_{xx}{\rm{\Psi }}+i\gamma {\rm{\Psi }}+\mathrm{(1}-i{\rm{\Gamma }})|{\rm{\Psi }}{|}^{2}{\rm{\Psi }}\mathrm{.}$$

Here *t* is time, *x* is the angular variable varying between 0 and 2*π*, and Ψ is the complex field, whose physical meaning depends on the chosen physical setting, and which is considered subject to the cyclic boundary conditions: Ψ(*x*, *t*) = Ψ(*x* + 2*π*, *t*).

Equation (1) has a simple solution

2 $${\rm{\Psi }}=\sqrt{\gamma /{\rm{\Gamma }}}\,{e}^{i\kappa x-i(\gamma /{\rm{\Gamma }}+{\kappa }^{2})t}$$

where *κ* is an integer number due to the quantization condition imposed by the ring geometry. One can readily verify that the solution (2) is linearly stable, and hence it is an attractor of Eq. (1). Let us mention that the field in a ring shaped waveguides subject to localized loss^28^, as well as gain and loss^29^, were already studied in the similar context.

**Figure 1:**
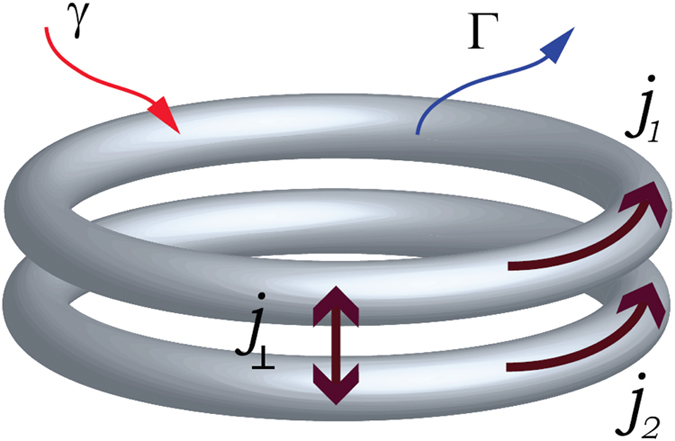
Schematic view of currents in the double ring structure with linear gain *γ* and nonlinear loss Γ.

It turns out that plane wave solutions, similar to the field (2) obtained for a single ring shaped waveguide with linear gain and nonlinear dissipation, may lead to quite rich dynamics, if more than one ring waveguides are coupled to each other. In this paper we report such dynamics in the case of two coupled rings, as schematically illustrated in Fig. 1.

Before going into details, we comment on the terminology used below. For the applications mentioned above the field Ψ (or the fields Ψ_1,2_ explored below) represents only angular and temporal, i.e. (*x*, *t*), dependence while the total field (either macroscopic parameter in the case of BECs or the electric field in the case of optical waveguides) depends on all three spatial coordinates. The respective three-dimensional distributions may obey phase singularity in the center of the ring (this is, in particular, the case of circular currents studied below). Thus, a solution of the reduced quasi-one-dimensional model (1) [or of the respective two-component model (4) studied in this paper] can be viewed as vortex solutions of the full field in the three-dimensional space. In this interpretation the parameter *κ* in (2) is identified as topological charge of the vortex. Indeed, for a field distribution whose phase in the cylindrical coordinates with the *z*-axis orthogonal to the ring-waveguide, depends only on the angle *ϕ*, the topological charge is computed as

3 $$\kappa =\frac{1}{2\pi }{\oint }_{Z}\nabla {\rm{\arg }}({\rm{\Psi }})\cdot d{\rm{l}}=\frac{1}{2\pi }{\int }_{0}^{2\pi }\frac{\partial }{\partial \varphi }{\rm{\arg }}({\rm{\Psi }})d\varphi \mathrm{.}$$

Here *Z* is a contour surrounding the phase singularity along the waveguide center. Since in the quasi-one-dimensional system (1) the angle is denoted by *x*, we conclude that a solution with ∼*e* ^*iκx*^ has the topological charge *κ*. Thus, in the following sections to distinguish currentless from current bearing states the latter will be referred to as vortices (by analogy with the terminology accepted, say in the optics of discrete vortices^30^).

The nonlinear dynamics inside each of and between the ring-shaped waveguides is characterized by flows, of either particle or energy, depending on application. The flows along waveguides may be different from the conventional currents of vortex states characterized exclusively by the vorticity. In particular the flows exist not only along each of the ring but also between the rings (see Fig. 1). It is the purpose of this paper to investigate these flows in the double-ring resonator. We concentrate on solutions that are stationary and allow for the constant flow between rings. We only concisely describe non-stationary solutions (see Sec. 2.4), although it is very interesting and challenging task. We discovered that the system under study experience dynamics reminding chaotic one, with rather reach structure of attractors, limit tori, exploding solitons and many other phenomena characteristic for chaos. This aspect is briefly mentioned here, but it deserves separate publication and will be presented in details elsewhere.

Now we turn to the model of the double-ring resonator (see Fig. 1) which includes dispersion, de-focusing (or repulsive) self-phase modulation, linear gain and nonlinear loss [cf. Eq. (1)]

4 $$\begin{array}{c}i{\partial }_{t}{{\rm{\Psi }}}_{1}=-{\partial }_{xx}{{\rm{\Psi }}}_{1}+i\gamma {{\rm{\Psi }}}_{1}+\mathrm{(1}-i{\rm{\Gamma }})|{{\rm{\Psi }}}_{1}{|}^{2}{{\rm{\Psi }}}_{1}+c{{\rm{\Psi }}}_{2}\\ i{\partial }_{t}{{\rm{\Psi }}}_{2}=-{\partial }_{xx}{{\rm{\Psi }}}_{2}+i\gamma {{\rm{\Psi }}}_{2}+\mathrm{(1}-i{\rm{\Gamma }})|{{\rm{\Psi }}}_{2}{|}^{2}{{\rm{\Psi }}}_{2}+c{{\rm{\Psi }}}_{1}\mathrm{.}\end{array}$$

The coupling is characterized by the (positive) constant *c*. As mentioned above, here we impose cyclic boundary conditions: Ψ_*α*_(*x*, *t*) = Ψ_*α*_(*x *+ 2*π*, *t*) (*α* = 1, 2). We mention that the system (4) can be viewed as a simplified model, whose extension accounting for quintic nonlinearity and diffusion was introduced and numerically studied in ref. 31 subject to zero boundary conditions. Later a similar model on the whole line^32^, but with one of the equations linear was considered in the context of plasmon solitons in a layered dielectric-metal geometry^33^.

All throughout this paper we will use the current densities along each ring (illustrated in Fig. 1):

5 $${j}_{\alpha }(x,t)=\frac{1}{2i}({{\rm{\Psi }}}_{\alpha }^{\ast }\frac{\partial {{\rm{\Psi }}}_{\alpha }}{\partial x}-{{\rm{\Psi }}}_{\alpha }\frac{\partial {{\rm{\Psi }}}_{\alpha }^{\ast }}{\partial x}),\quad \alpha =\mathrm{1,}\,2$$

the current density between the two rings

6 $${j}_{\perp }(x,t)=\frac{c}{2i}({{\rm{\Psi }}}_{1}^{\ast }{{\rm{\Psi }}}_{2}-{{\rm{\Psi }}}_{1}{{\rm{\Psi }}}_{2}^{\ast }),$$

and total current between the two rings

7 $${J}_{\perp }(t)={\int }_{0}^{2\pi }{j}_{\perp }(x,t)dx\mathrm{.}$$

Below we will show that while the simplest solutions obeying well defined symmetry posses zero transversal flows: ${j}_{\perp }=0$, stationary asymmetric solutions may have nonzero constant flows between the rings. What is even more striking, there are states where flows are modulated along the rings (showing a kind of symmetry breaking).

### Homogeneous states

#### Stationary solutions

We are primarily interested in stationary solutions and start with homogeneous states, which can be described by [cf. Eq. (2)]

8 $${{\rm{\Psi }}}_{1}={\rho }_{1}{e}^{i(\kappa x-\mu t+\theta )},\quad {{\rm{\Psi }}}_{2}={\rho }_{2}{e}^{i(\kappa x-\mu t-\theta )}\mathrm{.}$$

Here *ρ*_1,2_ are the moduli of the wavefunctions which have a constant phase mismatch between the components 2*θ*, and common chemical potential (frequency or propagation constant in the optical context). We also included vortex solutions accounted for by the phase factor *κx*. After substituting the ansatz (8) into Eq. (4) we obtain the relation among the parameters

9 $$({\rho }_{1}^{2}-{\rho }_{2}^{2})(\mu -{\kappa }^{2}-{\rho }_{1}^{2}-{\rho }_{2}^{2})=0.$$

The last formula allows for classification of solutions into *four* branches.

**Figure 2:**
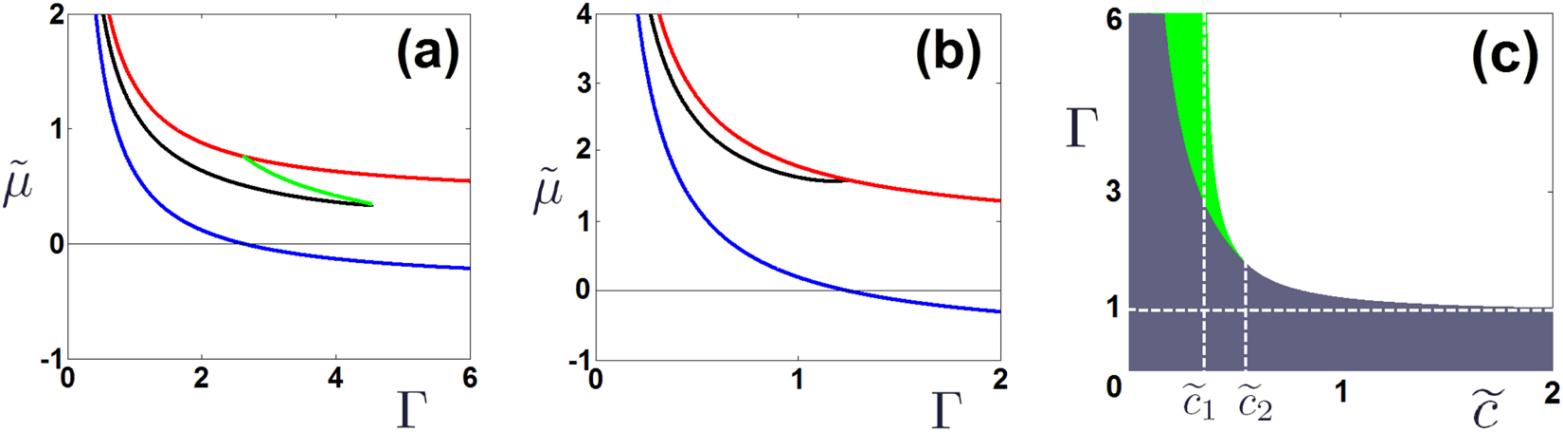
(**a**,**b**) Branches of the stationary solutions in the parameter plane $(\tilde{\mu },{\rm{\Gamma }})$. In all panels red and blue lines correspond to symmetric, ${\tilde{\mu }}_{+}=1/{\rm{\Gamma }}+\tilde{c}$, and antisymmetric, ${\tilde{\mu }}_{-}=1/{\rm{\Gamma }}-\tilde{c}$ states; green and black curves correspond to asymmetric modes with ${\tilde{\mu }}_{+}^{(a)}$ and ${\tilde{\mu }}_{-}^{(a)}$. (**a**) Case $\tilde{c}=0.38 < {\tilde{c}}_{2}$ where one can find two different asymmetric modes ${\mu }_{\pm }^{(a)}$; (**b**) Case $\tilde{c}=0.8 > {\tilde{c}}_{2}$ where the system supports only one asymmetric mode ${\mu }_{-}^{(a)}$. Panel (c) shows the regions in the plane $({\rm{\Gamma }},\tilde{c})$ where the system supports asymmetric modes. In the green area there are two asymmetric modes, in the gray area only one type of modes ${\mu }_{-}^{(a)}$ exists. The upper border of two areas is the curve ${{\rm{\Gamma }}}_{{\rm{cr}}}(\tilde{c})$ while the lower border of the green area is the curve ${{\rm{\Gamma }}}_{{\rm{bf}}}(\tilde{c})$.

The first two branches are obtained when the first factor in Eq. (9) vanish, i.e. when *ρ*_1_ = *ρ*_2_. This yields *symmetric θ* = 0 and *antisymmetric θ* = *π*/2 solutions. It is easy to find the amplitudes of both solutions ${\rho }_{1}={\rho }_{2}=\sqrt{\gamma /{\rm{\Gamma }}}$ as well as the chemical potentials: *μ*_±_ = *γ*/Γ + κ^2^ ± *c*, where + and − stand for symmetric and antisymmetric states, respectively. For the future consideration it is convenient to introduce the renormalized chemical potential $\tilde{\mu }=(\mu -{\kappa }^{2})/\gamma$, the renormalized coupling constant $\tilde{c}=c/\gamma$. To simplify analytic form of our formulas in this paragraph we will use renormalized chemical potential defined for symmetric *μ*_+_ (antisymmetric *μ*_−_) states by ${\tilde{\mu }}_{\pm }=1/{\rm{\Gamma }}\pm \tilde{c}$. The brunches of solutions described above are shown in Fig. 2, represented by red and blue lines.

Next we consider the second factor in Eq. (9). It gives us two branches of *asymmetric* solutions. In this case the (renormalized) chemical potential can be written down as

10 $${\tilde{\mu }}_{\pm }^{(a)}=\frac{3{{\rm{\Gamma }}}^{2}-1\pm \sqrt{{({{\rm{\Gamma }}}^{2}+\mathrm{1)}}^{2}-8{\tilde{c}}^{2}{{\rm{\Gamma }}}^{2}({{\rm{\Gamma }}}^{2}-\mathrm{1)}}}{2{\rm{\Gamma }}({{\rm{\Gamma }}}^{2}-\mathrm{1)}}\mathrm{.}$$

The explicit expressions for the field amplitudes of the asymmetric modes now read

11 $$\begin{array}{l}{\rho }_{\mathrm{1,}\pm }^{2}=\frac{\gamma }{2}({\tilde{\mu }}_{\pm }^{(a)}-\sqrt{\frac{2{\tilde{\mu }}_{\pm }^{(a)}}{{\rm{\Gamma }}}-{[{\tilde{\mu }}_{\pm }^{(a)}]}^{2}}),\\ {\rho }_{\mathrm{2,}\pm }^{2}=\frac{\gamma }{2}({\tilde{\mu }}_{\pm }^{(a)}+\sqrt{\frac{2{\tilde{\mu }}_{\pm }^{(a)}}{{\rm{\Gamma }}}-{[{\tilde{\mu }}_{\pm }^{(a)}]}^{2}}),\end{array}$$

and the phase mismatches are given by

12 $${\cos }^{2}\mathrm{(2}{\theta }_{\pm })=\frac{{\tilde{\mu }}_{\pm }^{(a)}}{2{\tilde{c}}^{2}}({\tilde{\mu }}_{\pm }^{(a)}-\frac{1}{{\rm{\Gamma }}})\mathrm{.}$$

Notice that symmetric and antisymmetric solutions depend only on the ratio *γ*/Γ and the coupling *c*, while asymmetric solutions, depend on all three parameters separately.

Next we find regions of parameters *γ*, $\tilde{c}$ and Γ where the asymmetric modes exist. The phase mismatch *θ*_±_ must be real and both amplitudes *ρ*_1,±_, *ρ*_2,±_ must be positive thus we have restrictions for chemical potential

13 $$1/{\rm{\Gamma }}\le {\tilde{\mu }}_{\pm }^{(a)}\le 2/{\rm{\Gamma }}\mathrm{.}$$

Besides, the right hand side of Eq. (10) must be real so there is a critical value of renormalized coupling ${\tilde{c}}_{1}=1/\sqrt{8}$ below which the system supports two asymmetric modes (for all value of Γ), and above it asymmetric modes survive only if

14 $${\rm{\Gamma }} < {{\rm{\Gamma }}}_{{\rm{cr}}}={[4\tilde{c}(\sqrt{1+{\tilde{c}}^{2}}-\tilde{c})-1]}^{-\mathrm{1/2}}\mathrm{.}$$

The two branches of the asymmetric solutions are shown in Fig. 2a,b. The upper branch ${\tilde{\mu }}_{+}^{(a)}$ exists only on the finite interval ${{\rm{\Gamma }}}_{{\rm{bf}}}\le {\rm{\Gamma }}\le {{\rm{\Gamma }}}_{{\rm{cr}}}$ where ${{\rm{\Gamma }}}_{{\rm{bf}}}=1/\tilde{c}$ [the green line in Fig. 2a]. At Γ = Γ_bf_ the asymmetric branch ${\tilde{\mu }}_{+}^{(a)}$ bifurcates from the symmetric branch *μ*_+_. The chemical potentials of the both branches at this point are equal to the right boundary 2/Γ_b*f*_ in (13) and the amplitudes of field components in the mode are given by ${\rho }_{\mathrm{1,}\pm }={\rho }_{\mathrm{2,}\pm }=\sqrt{\gamma /{{\rm{\Gamma }}}_{{\rm{bf}}}}$. At the critical point Γ = Γ_cr_ we observe coalescence of the two branches of the asymmetric modes.

At the left boundary (${\tilde{\mu }}^{(a)}=1/{\rm{\Gamma }}$) the symmetric and asymmetric states do not meet. Their chemical potentials asymptotically approach each other when Γ → 0 (then ${\tilde{\mu }}_{-}^{(a)}\sim {\tilde{\mu }}_{\pm }\to \mathrm{1/}{\rm{\Gamma }}$) but the other parameters of the wavefunctions remain different. At this limit, the asymmetric mode becomes highly asymmetric with *ρ*_1,−_ → 0 and ${\rho }_{\mathrm{2,}-}\to \sqrt{\gamma /{\rm{\Gamma }}}$, while the wavefunction of a symmetric mode is ${\rho }_{1}={\rho }_{2}=\sqrt{\gamma /{\rm{\Gamma }}}$.

From the above analysis it follows that the upper asymmetric branch disappears as the interval of its existence shrinks to a point. This occurs at Γ_bf_ = Γ_cr_, which gives us the critical value of renormalized coupling ${\tilde{c}}_{2}=1/\sqrt{3}$. Therefore, in the range $\tilde{c} < {\tilde{c}}_{2}$ we observe two stationary asymmetric modes with ${\tilde{\mu }}_{\pm }^{(a)}$ [Fig. 2a] while in the range $\tilde{c} > {\tilde{c}}_{2}$ we have only one mode ${\tilde{\mu }}_{-}^{(a)}$ [Fig. 2b].

In Fig. 2c we show the domains of the existence of the asymmetric modes. As we mentioned above two asymmetric modes always exist for weak coupling. When this coupling becomes stronger, $\tilde{c} > {\tilde{c}}_{1}$, such two asymmetric modes can be still excited if the nonlinear absorption is nonzero but is not too strong (the green area). However, when the coupling exceeds the critical point, $\tilde{c} > {\tilde{c}}_{2}$, only one asymmetric mode can exist for small values of Γ (gray domain).

Turning now to the current densities, we first notice that the longitudinal currents defined by Equation (5), are trivially determined by the topological charge *κ*. The transverse currents (6) depend of the type of the solution. For vortex, symmetric and antisymmetric solutions we have zero current, ${J}_{\perp }=0$.

For the case of asymmetric modes we calculate

15 $${J}_{\perp }^{(\pm )}=\pi {\gamma }^{2}\sqrt{{\tau }_{\pm }\mathrm{(2}{\tilde{c}}^{2}-{\tau }_{\pm })}$$

where ${\tau }_{\pm }={\tilde{\mu }}_{\pm }^{(a)}({\tilde{\mu }}_{\pm }^{(a)}-\mathrm{1/}{\rm{\Gamma }})$. Since the two rings are identical, the existence of the transverse currents represents the spontaneous *symmetry breaking*.

**Figure 3:**
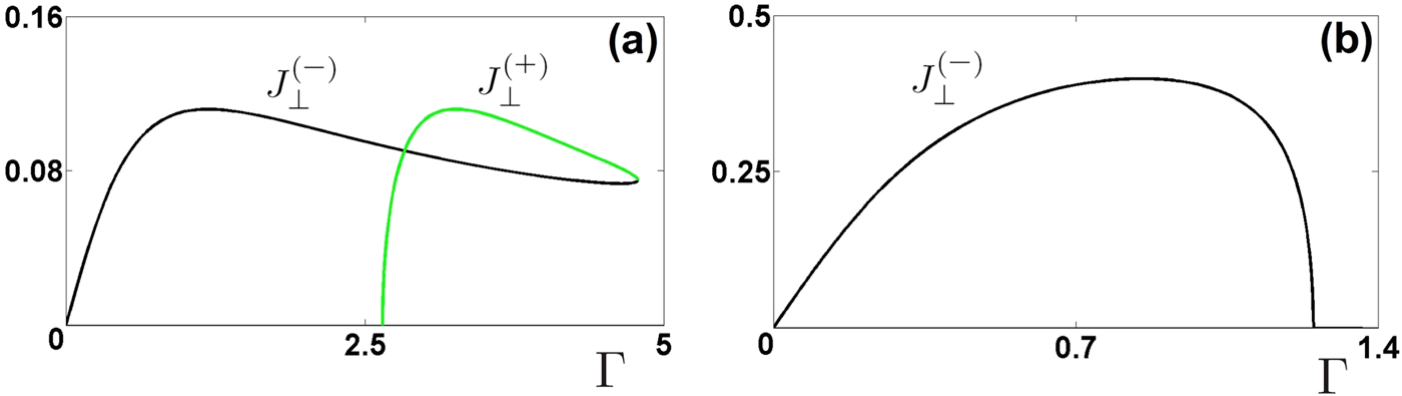
Transverse currents between the rings for the asymmetric modes ${J}_{\perp }^{(\pm )}({\rm{\Gamma }})$; (**a**) the two currents in the case of $\tilde{c}=0.38$ (green and black lines correspond to the upper ${\tilde{\mu }}_{+}^{(a)}$ and lower ${\tilde{\mu }}_{-}^{(a)}$ modes); (**b**) the current of the lower asymmetric mode ${J}_{\perp }^{(-)}$ in the case of $\tilde{c}=0.8$. In both cases *γ* = 0.5.

In Fig. 3 we show the transverse currents as function of Γ for two typical situations where the system supports either two or one asymmetric mode [see also the panels (a) and (b) in Fig. 2]. Several features of the curves shown in the figure are to be emphasized. First, the transverse current densities are strongly monotonic functions of the nonlinear absorption. They increase with Γ achieving maxima (different modes at different absorptions) and then decay until the point where upper and lower branches coalesce. Second, when both, upper and lower, modes exist they feature equal transverse currents at some intermediate absorption (crossing of black and green lines) rather than only in the coalescence point at Γ_c*r*_ (where the two branches coalesce).

#### Linear stability analysis

Experimental feasibility and utility of the solutions introduced above is determined by their stability. To address this issue we examine the linear stability of the modes (8). As it is customary, we consider the perturbed states in form of (*α* = 1, 2)

16 $${\tilde{{\rm{\Psi }}}}_{\alpha }={e}^{i(\kappa x-\mu t)}[{\rho }_{\alpha }{e}^{{(-\mathrm{1)}}^{\alpha }i\theta }+{e}^{\lambda t}{U}_{\alpha }(x)+{e}^{{\lambda }^{\ast }t}{V}_{\alpha }^{\ast }(x)],$$

where *λ* is the spectral parameter and |*U*|, |*V*| ≪ *ρ*_*α*_. The solution is unstable if Re (*λ*) > 0. Substituting (16) in Equation (4), linearizing with respect to the perturbations taken in the form (*U*, *V* ∼ *e* ^*iκx*^) we obtain the linear eigenvalue problem

17 $$\hat{L}{({U}_{1},{V}_{1},{U}_{2},{V}_{2})}^{T}=\lambda {({U}_{1},{V}_{1},{U}_{2},{V}_{2})}^{T}\mathrm{.}$$

Here *T* stands for transpose matrix, and the operator $\hat{L}$ is the constant matrix

18 $$\hat{L}=(\begin{array}{llll}{\chi }_{1} & {\beta }_{1}^{\ast } & -ic & 0\\ {\beta }_{1} & {\chi }_{1}^{\ast } & 0 & ic\\ -ic & 0 & {\chi }_{2} & {\beta }_{2}^{\ast }\\ 0 & ic & {\beta }_{2} & {\chi }_{2}^{\ast }\end{array})$$

with the entries (*α* = 1, 2).

19 $$\begin{array}{c}{\chi }_{\alpha }=i(\mu -{(k+\kappa )}^{2}-2{\rho }_{\alpha }^{2})+\gamma -2{\rm{\Gamma }}{\rho }_{\alpha }^{2},\\ {\beta }_{\alpha }={\rho }_{\alpha }^{2}{e}^{{(-\mathrm{1)}}^{\alpha }2i\theta }(i-{\rm{\Gamma }}\mathrm{).}\end{array}$$

Thus the matrix $\hat{L}$ depends only on the combination *k* +* κ* (rather than on *k* and *κ* separately). This means that the linear stability of the modes with different vorticities *κ* is the same as the stability of the modes with the same densities and chemical potentials, but having zero vorticity. Therefore below we concentrate only on the linear stability of the modes without vorticity (notice that this is not generalized to the dynamics of inhomogeneous solutions, as it is discussed below).

The straightforward analysis of the linear stability shows that the antisymmetric modes [*θ* = *π*/2 in (8)] are stable for all values of the parameters. On the other hand symmetric states are mostly unstable, and small perturbation may lead to interesting dynamics and discovery of new, both stationary and non-stationary states. In the next subsection we present the results of numerical studies of evolution of symmetric states subject to small perturbation.

#### Stability of symmetric states

Investigation of the stability of symmetric modes [*θ* = 0 in Eq. (8)] provides very rich variation of various types of dynamics and can be used as a tool to find new (not necessary symmetric) stable solutions. Linear stability analysis predicts the windows of instability of modes with wave vectors satisfying the relation

20 $$2(c-\frac{\gamma }{{\rm{\Gamma }}}) < {k}^{2} < 2c\mathrm{.}$$

**Figure 4:**
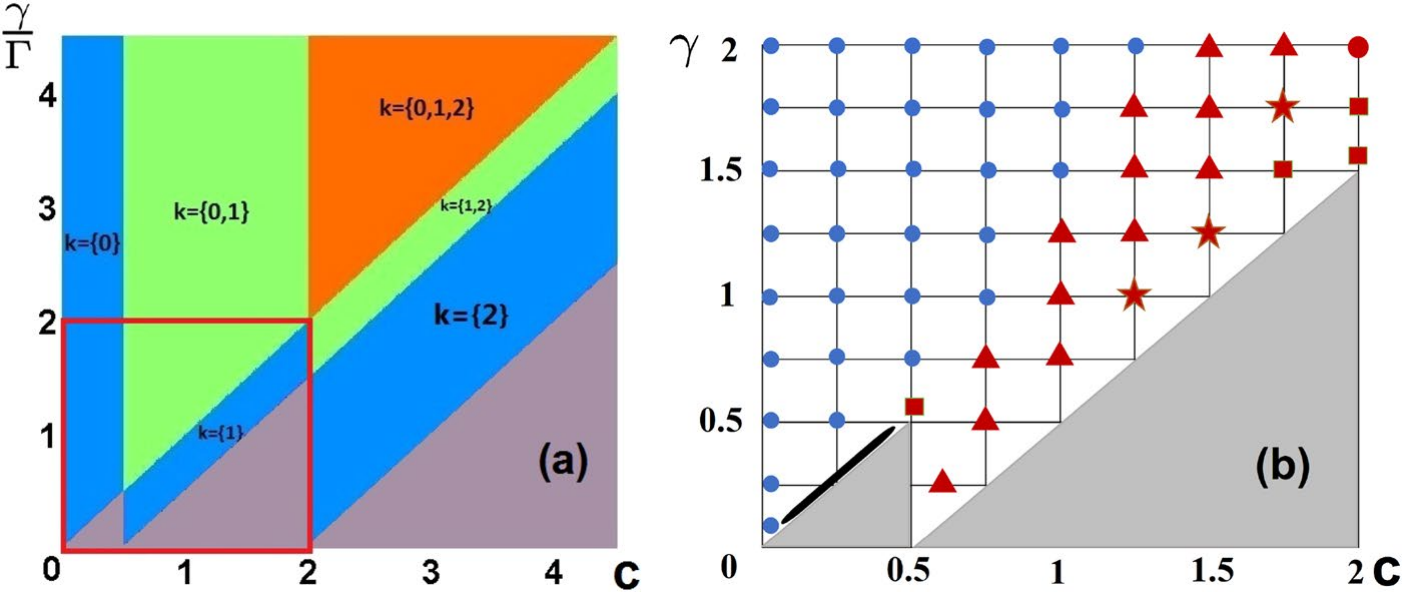
(**a**) Domains of instability. In each domain we indicate wave-vectors of unstable modes obtained for the symmetric states found using formula (20). Different colors correspond to different numbers of unstable modes. The gray regions correspond to the stable solutions. The red square outlines the domain in which evolution of initially perturbed states are studied and discussed in second panel. (**b**) Final (destination) states reached by direct propagation starting from initially perturbed symmetric state. The grey areas correspond to stable regimes, blue small circles correspond to antisymmetric states reached as final destination, red triangles out-of-phase vortices (with *κ* = 1). Red stars represent limit cycle, red squares - inhomogeneous solutions and red big circle in the upper right corner illustrate “chaotic” motion around fixed point and black stripe corresponds to asymmetric states. Points on the left axis correspond to very week coupling *c* = 0.01. For details see section 2.3.

The schematic view of instability domains with indication of unstable modes (note that *k* vectors are quantized in our system) is illustrated in Fig. 4a. Notice that while the areas of the stability domains increase with the strength of the coupling *c* for lower values of *γ*/Γ, for larger values of the last parameter increase of the coupling may be destabilizing and lead to increasing the number of unstable modes. For any given coupling coefficient the system becomes linearly unstable if the gain is large enough.

Since the coupled-rings system at hand is dissipative, evolution of the unstable states is expected to end up at an attractor solution. To explore possible dynamical regimes we checked numerically that when the symmetric state, described above, is initially perturbed, it may evolve to several different attractors, depending on the system parameters *γ*/Γ and *c*. Our results are collected in Fig. 4b, where we show the points in the parameter space at which the initial modes were chosen to propagate (the black line grid is for the sake of convenience only). Notice that points are marked with different symbols which characterize the type of dynamics that one obtain using (slightly perturbed - we always used the same perturbation proportional to cos(3*x*)) symmetric states corresponding to the appropriate values of *c* and *γ* as initial conditions (in these simulations we fixed Γ = 1). Below we present detailed description of the dynamics divided into several classes.

Regions of stability of symmetric states and marked them with grey background (see formula instabilitymodes). When one starts from the points marked with blue circles (and apply small initial perturbation) the dynamics leads to stable antisymmetric states, corresponding to the appropriate values of *c* and *γ*. If the evolution starts from any of the points corresponding to red triangle, it leads to out-of-phase vortices with *κ* = 1 (notice that we are dealing with open system and topological charge is not a conserved quantity). But on close inspection Fig. 4b reveals more interesting features. In the region near the lower left corner of the diagram we identified a whole region of states that, being initially perturbed, propagate towards asymmetric states. It is not clear why they only form a narrow band in certain region of Fig. 4b, but we checked that their parameters can be described by formulas (10)–(12) and they are stable according to the linear stability analysis. It is located on the border of stable region for symmetric states, close to the origin in Fig. 4b. We carefully investigated other border lines of grey regions in Fig. 4b but we did not find any asymmetric solutions, that were stable.

We also identified inhomogeneous states as a final destination and we marked them with red squares. They are described in the separate paragraph below. Notice that we do not have analytical formulae for inhomogeneous stable solutions and our study, through dynamics is the only way to find and analyze them.

Besides these stable destinations, evolution can lead to even more complicated phases, marked with red stars (we call them limit cycle) and red circle corresponding to irregular motion around fixed point. To describe this type of dynamics in details we designated a separate section.

Finally we observe that the distribution of the final states on the diagram in Fig. 4b remotely resemble distribution of the unstable regions in the diagram in Fig. 4a. In particular, whenever the homogeneous perturbation with *k* = 0 represents an unstable mode, the final state is the homogeneous antisymmetric state (blue circles), while when *k* = 1 is the lowest unstable mode the attractor in the most of cases consist of out-of-phase vortices with the topological charge *κ* = 1. The most strong deviations from the behavior (shown by “star” and “circle” states) mention above is observed in the vicinity of the boundaries between unstable domains of different types in Fig. 4a.

#### 2.4 Irregular behavior

**Figure 5:**
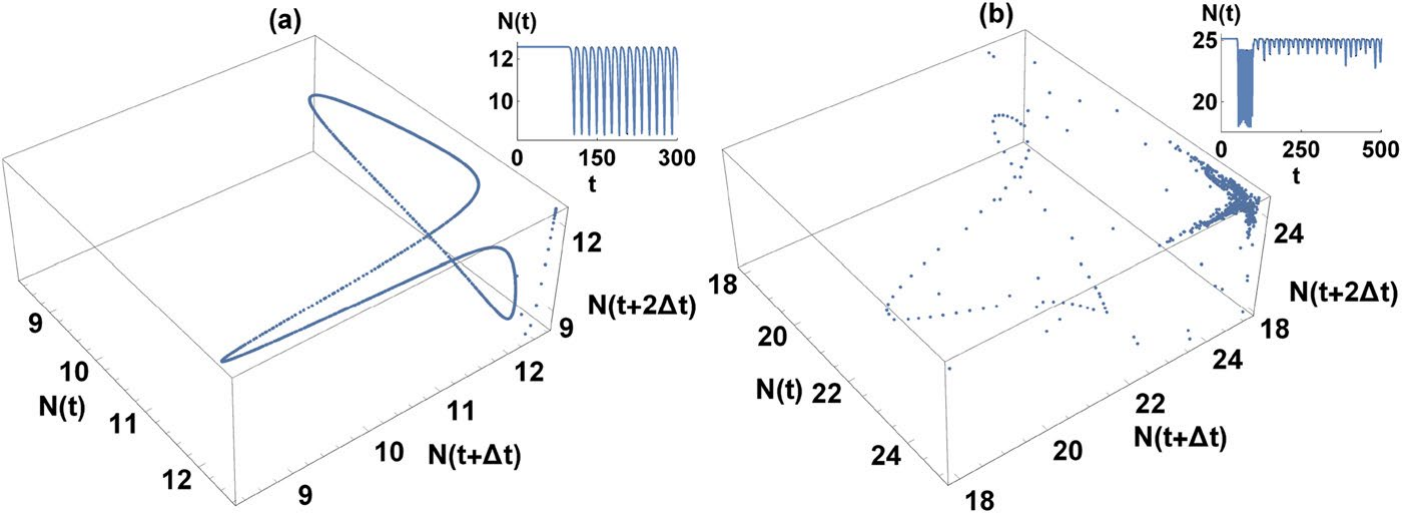
Phase portraits illustrating dynamics of limit circle (**a**) (Γ = *γ* = 1, *c* = 1.25) and chaos (**b**) (Γ = 1, *γ* = *c* = 2). Small frames show corresponding time dependence of total norm in both rings.

In addition to the regular stable solutions emerging in the evolution starting from symmetric states, in the numerical simulations we identified also attractors in the form of limit cycle, and “chaotic” motion. The are presented in Fig. 4b as red stars (limiting-cycle-like solutions) and red circle in the upper right corner (irregular, “chaotic” motion around fixed point). Characteristics of such solutions can be better understood by inspecting their phase portraits in Fig. 5, inspired by Takens and Mane embedding theorem, building from scalar quantity - total norm - multidimensional phase space consisting of *N*(*t*), *N*(*t* + Δ*t*) and *N*(*t* + 2Δ*t*), with the delay Δ*t* arbitrary. In panel (a), we show typical limit cycle behavior. The total norm (in the small frame) is oscillating in the regular manner around the value of 4*πγ*/Γ. In the panels (b) we present the dynamics corresponding to the red circle (at the point with value of parameters are *c* = 2 and *γ* = 2 in Fig. 4b), in which in the right corner we observe chaotic attractor. The vale of the norm stays close to 4*πγ*/Γ, but the trajectory jumps away from central point in Fig. 5 and after few irregular oscillations comes back. This kind of behavior also appears in the regions corresponding to higher values of *γ* and *c*.

### Inhomogeneous States

In some region of parameter space, perturbed unstable symmetric states tend to inhomogeneous solutions. They are marked with red squares in Fig. 4b. These solutions can not be obtained analytically, but we checked their stability in real time propagation over very long time, of order of 2000 in dimensionless units (twice as much as was necessary to reach steady state in any of our simulations). Within the limited range of parameters investigated in present study we found just a few regions where inhomogeneous states occur, but our evidence is sufficient to formulate several general observations.

**Figure 6:**
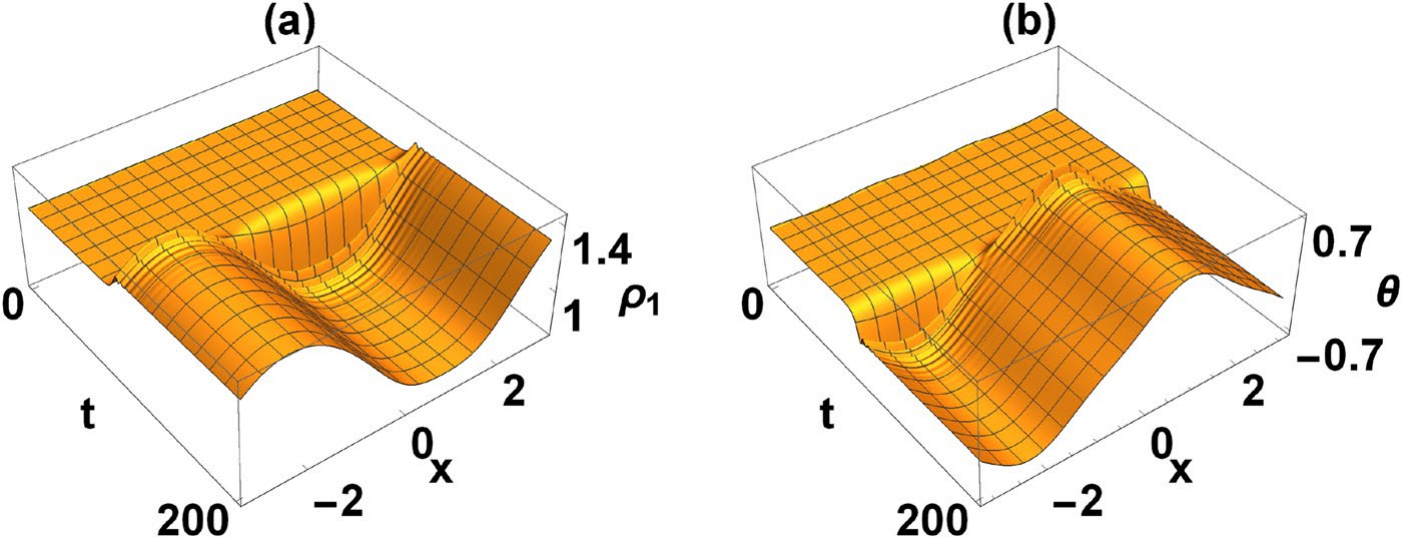
Dynamics of unstable symmetric state (with *κ* = 0). The final destination is an inhomogeneous state. Value of parameters are Γ = 1, *γ* = 1.5 and *c* = 1.75. (**a**) Amplitude of wavefunction in the first ring *ρ*_1_(*x*, *t*) (here we show the first ring only; the final form of both wavefunctions are shown in Fig. 7a); (**b**): relative phase *θ*(*x*, *t*).

Inhomogeneous states are particularly interesting because they admit permanent, inhomogeneous current flows between rings, i.e. they exhibit symmetry breaking both in longitudinal and in transversal directions. In the numerical cases explored here such currents were (almost) periodically modulated (note that constant current is also present in asymmetric solutions, but it is homogenous). We also noticed, when we starting from vortex states, one can reach inhomogeneous states with currents modulated in time. In Fig. 6 we present an example of the dynamics (time evolution) starting with symmetric state with Γ = 1, *γ* = 1.5 and *c* = 1.75, where we can clearly see the transition to the final inhomogeneous state. In the panels (a) we see the modulus of the wavefunction, in panel (b) the relative phase. In both cases after some transient dynamics steady state is reached, but the location of its maximum is very sensitive to the type of perturbation that is initially applied. It is due to the spontaneous translational symmetry breaking that we experience here.

**Figure 7:**
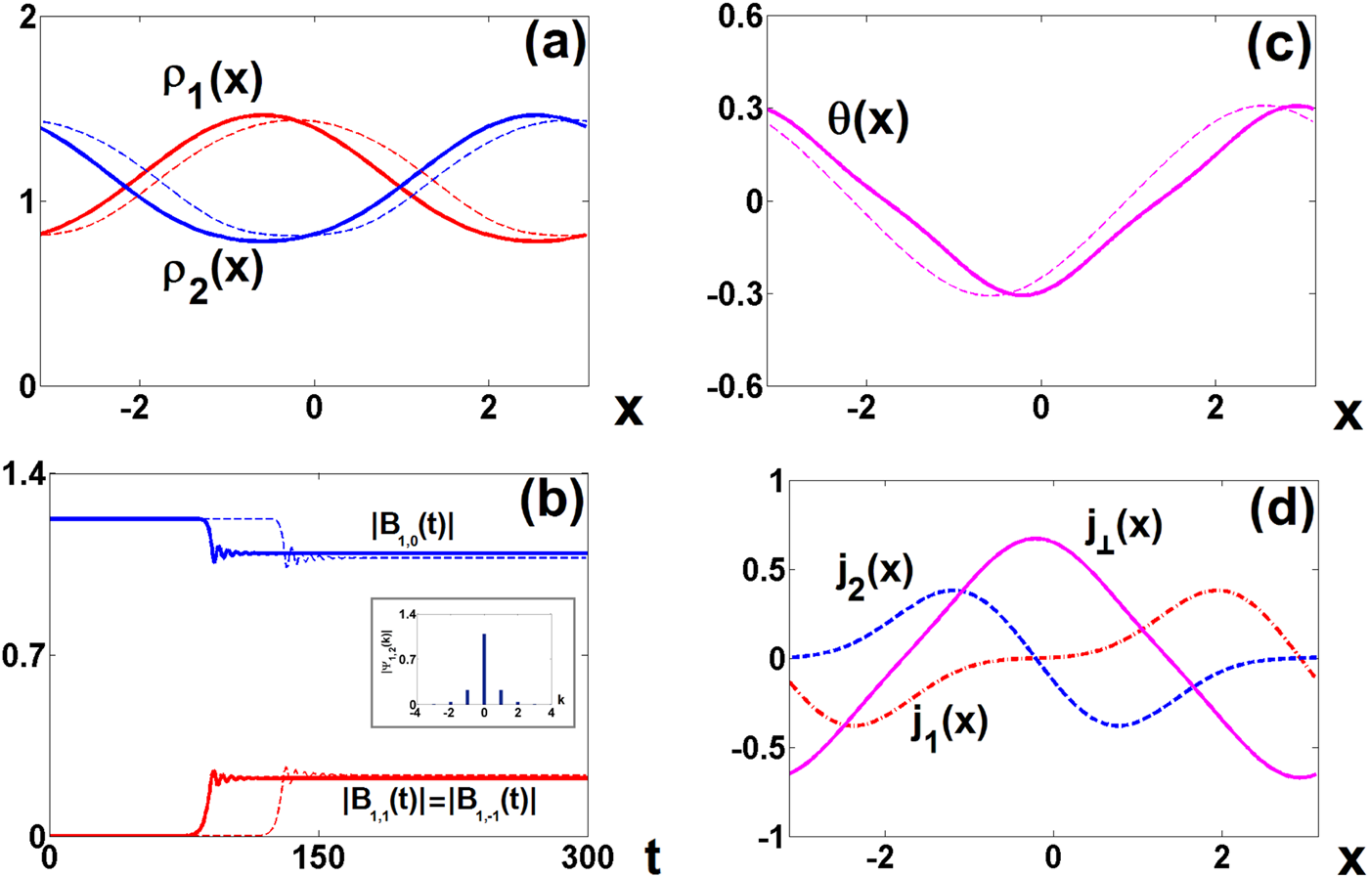
Inhomogeneous state obtained from the evolution of an unstable symmetric state showing in Fig. 6a Amplitudes of wavefunctions *ρ*_1,2_(*x*); (**b**) Time evolution of main spatial Fourier components |*B*_*α*,*k*_(*t*)| and Fourier spectrum of the inhomogeneous state (inset); (**c**) Relative phase *θ*(*x*). In the panels (**a**–**c**), the solid curves represent full numerical simulation while dashed curves correspond to Galerkin approximation accounting for the modes *k* = 0, ±1 according to (22). (**d**) Currents: within rings *j*_1,2_(*x*) (dotted and dash curves) and current between rings ${j}_{\perp }(x)$ (solid curve).

In Fig. 7 we show some characteristics of inhomogeneous state at the end of evolution presented in Fig. 6. In the panel (a) we plot the moduli of both wavefunctions *ρ*_1_ and *ρ*_2_ as a function of the position. We observe clear modulation with the period equal to one. This is confirmed when we look at the Fourier decomposition. Besides constant component there is one dominant contribution from *k* = ±1, and higher components are (almost) negligible. Additionally we show relative phase between Ψ_1_ and Ψ_2_ in panel (c) and the structure of currents flowing in and between rings in panel (d). We investigated the structure of all inhomogeneous states that appeared as a final destination in our dynamics starting from symmetric state and we checked that they all have similar features. Furthermore, we also checked that it is possible to obtain inhomogeneous solutions starting from unstable in-phase vortex states. In this case the Fourier spectrum is also limited, but shifted to include the initial topological charge.

In all the cases one can describe both wavefunctions using the Fourier expansion

21 $${{\rm{\Psi }}}_{\alpha }(x,t)={e}^{i(\kappa x-\mu t)}\sum _{n=-\infty }^{\infty }{B}_{\alpha ,n}(t){e}^{inx}$$

where *α* refers to the number of the ring (*α* = 1, 2) and *κ* to the topological charge. In our simulations when we reach inhomogeneous state complex coefficients *B*_*α*,*n*_ satisfy certain symmetry conditions. In particular, we found that always B_1,*n*_ = (−1)^*n*^ *B*_2,*n*_. Additionally we noticed that when we start with zero topological charge (unstable symmetric) state, the modulus of the Fourier spectrum of the final destination inhomogeneous state, as we can verify in Fig. 7, is symmetric with respect to the origin: |*B*_*α*,*n*_| = |*B*_*α*,−*n*_|. If we start from unstable in-phase vortex state with topological charge equal to *κ* and end up in the inhomogeneous state, the relation is modified to |*B*_*α*,*n*_| = |*B*_*α*,−*n*+2*κ*_|.

Even sufficiently complex dynamics of vortices, may be described with relatively high precision^34^, using the so-called Galerkin approximation, i.e. the variational-type ansatz (21) where sum is computed only over a few lowest harmonics. In all the cases marked on the Fig. 4b we checked that very good approximation is preserved if one truncates the sum in (21), by only three lowest modes with *n* = {0, ±1} (this is clearly seen in the inset of Fig. 7b). The coefficients *B*_*α*,*n*_(*t*) for the truncated sum satisfy the following dynamical equations

22 $$\begin{array}{c}i\frac{d{B}_{\mathrm{1,}n}}{dt}=(i\gamma +{(\kappa +n)}^{2}-\mu ){B}_{\mathrm{1,}n}+\mathrm{(1}-i{\rm{\Gamma }})\sum _{l=-1\,}^{1}\sum _{m=-1}^{1}{B}_{\mathrm{1,}l+m-n}^{\ast }{B}_{\mathrm{1,}l}{B}_{\mathrm{1,}m}+c{B}_{\mathrm{2,}n},\\ i\frac{d{B}_{\mathrm{2,}n}}{dt}=(i\gamma +{(\kappa +n)}^{2}-\mu ){B}_{\mathrm{2,}n}+\mathrm{(1}-i{\rm{\Gamma }})\sum _{l=-1\,}^{1}\sum _{m=-1}^{1}{B}_{\mathrm{2,}l+m-n}^{\ast }{B}_{\mathrm{2,}l}{B}_{\mathrm{2,}m}+c{B}_{\mathrm{1,}n}\mathrm{.}\end{array}$$

The results of the comparison of the direct numerical simulations with the Galerkin approximations are shown in panels (a)–(c) of Fig. 7. We observe that the dynamics governed by (22) reproduces the evolutions described by the full system. Possibly the most significant difference in the time delay of the instability in the Galerkin method with respect to the full model (cf. solid and dashed lines in Fig. 7b). This delay can be explained by the fact that the effectively initial perturbation of the full model possess much reacher than that of the Galerking approximation based only on the three central modes, and hence the instability growth in the full model is expected faster.

## Conclusions

In this work we reported very rich behaviors of a simple system consisting of two identical ring-shaped nonlinear waveguides in the presence of the linear gain and nonlinear dissipation. We have identified stable and unstable solutions. The former ones appear either symmetric or represent different types of the symmetry breaking, which can be expressed either in nonzero transversal currents (asymmetric solutions) or in inhomogeneous density distributions along the waveguides. Unstable solutions, being initially perturbed, can manifest different dynamics which ends up either in one of the stable state (homogeneous, vortex, asymmetric, or inhomogeneous) or display chaotic-like behavior.
